# Dynamic multiple-scattering treatment of X-ray absorption: Parameterization of a new molecular dynamics force field for myoglobin

**DOI:** 10.1063/1.5031806

**Published:** 2018-09-12

**Authors:** Giovanni Chillemi, Massimiliano Anselmi, Nico Sanna, Cristiano Padrin, Lodovico Balducci, Marco Cammarata, Elisabetta Pace, Majed Chergui, Maurizio Benfatto

**Affiliations:** 1 CINECA, SuperComputing Applications and Innovation Department, Via dei Tizii 6, 00185 Roma, Italy; 2 CNR-IBBE, Via Amendola 165, 70126 Bari, Italy; 3 Institute for Microbiology and Genetics, Georg-August-University Göttingen, Justus-von-Liebig-Weg 11, 37077 Göttingen, Germany; 4 CNR-NANOTEC (P.LAS.M.I. Lab), Via Amendola 122/D, 70126 Bari, Italy; 5 Université de Rennes 1, CNRS, Univ. Bretagne Loire, Institut de Physique de Rennes, UMR 6251, Rennes F-35042, France; 6 Laboratori Nazionali di Frascati, INFN- Via E. Fermi 44, 00044 Frascati, Italy; 7 Lab. of Ultrafast Spectroscopy (LSU) and Lausanne Centre for Ultrafast Science (LACUS), Ecole Polytechnique Fédérale de Lausanne, ISIC, FSB, Station 6, CH-1015 Lausanne, Switzerland

## Abstract

We present a detailed analysis of the X-ray absorption near-edge structure (XANES) data on the Fe K-edge of CO Myoglobin based on a combined procedure of Molecular Dynamics (MD) calculations and MXAN (Minuit XANes) data analysis that we call D-MXAN. The ability of performing quantitative XANES data analysis allows us to refine classical force field MD parameters, thus obtaining a reliable tool for the atomic investigation of this important model system for biological macromolecules. The iterative procedure here applied corrects the greatest part of the structural discrepancy between classical MD sampling and experimental determinations. Our procedure, moreover, is able to discriminate between different heme conformational basins visited during the MD simulation, thus demonstrating the necessity of a sampling on the order of tens of nanoseconds, even for an application such X-ray absorption spectroscopy data analysis.

## INTRODUCTION

X-ray absorption spectroscopy (XAS) is a powerful analytical technique to get structural and electronic information on the absorbing site of different types of matter, from biological systems to condensed materials. The low energy part of the XAS spectrum is called XANES (X-ray absorption near-edge structure) and is extremely rich of information: oxidation state, overall symmetry, distances, and angles between the atomic species around absorbing site can be derived from this part of the XAS spectra.^1^ In principle, an almost complete quantitative recovery of the geometrical structure within 6–7 Å from the absorber can be achieved from the XANES spectrum.^2^ In recent years, Benfatto and co-workers proposed a fitting procedure (called Minuit XANes, MXAN)) based on multiple-scattering (MS) theory which is able to extract local structural information around absorbing atoms from experimental XANES data.^3^ The MXAN method has been successfully used to analyze known and unknown systems, obtaining structural information comparable to X-ray diffraction and standard EXAFS analysis.^4–6^ In particular, the possibility of performing quantitative analysis on the XANES energy region is very relevant for metallo-protein systems where both the low S/N ratio and the weak scattering power of the light elements surrounding the metal site limit the energy range of the XAS data available for the analysis.

Myoglobin is a globular protein containing a heme prosthetic group that reversibly binds small ligands such as oxygen, carbon monoxide, and nitric oxide.^7^ For its small size, the relative structural stability, and the yet complex functional behavior, myoglobin is often used as model system in biophysics to study structure-dynamics-function relationships.^8^ Myoglobin docks the exogenous ligand in a cavity, the distal pocket, delimited mainly by hydrophobic residues and by the heme. Among the residues lining the distal pocket, the distal histidine plays a relevant role in fine-tuning the ligand affinities or in stabilizing the bound state of the ligand through a hydrogen bond with its N_ε_ proton.^9^ In addition, the distal histidine may adopt two distinct conformations, corresponding, respectively, to the closed and open state of the so-called “histidine-gate,” which may represent a short and quickly accessible escape route for the ligand.^10^

The myoglobin protein bonded to carbon monoxide (MbCO) has been studied for several years with many experimental and computational techniques, due to its important biological role.^11–13^ In particular, classic MD simulations have been applied to highlight, among others, the diffusion of small ligand in the hydrophobic cavities of the protein matrix, either in solution^14–18^ or in the crystal environment,^19^ the protein conformational transitions upon ligand photodissociation,^20,21^ and the spectroscopic properties of myoglobin or of the exogenous ligands inside its cavities.^22–25^

For that reason, it has been assisted at the developing of force fields for the description of the geometry and conformational dynamics of the heme cofactor, in different coordination states and in coordination with several ligands, in particular, with the carbon monoxide.^26–31^ However, the force field developed for the heme group still shows some limits. Among the big varieties of experimental data, the heme group has been fully described using the XAS technique at the iron K-edge.^4,32^ These experiments have put in evidence structural parameters such as the distance and angle between Fe and the CO molecule, which are not well reproduced by the classical force field.^4^

The combination of MXAN analysis and MD simulations (named D-MXAN analysis)^1,33^ can be a powerful tool to test in detail the classical force fields used in MD because it allows a quantitative comparison between calculation and experimental data. In the last years, the D-MXAN method has been successfully applied to aqueous ionic solutions^33–35^ and metallo-proteins.^36,37^

In the following, we report an iterative application of D-MXAN to the myoglobin protein bonded to carbon monoxide (MbCO) to evaluate the MD force field parameters and, when applicable, to refine them with the twofold objective of allowing a better knowledge of the MbCO structural/dynamic characteristics in solution and producing a more accurate MbCO force field for the community.

The experimental details and data normalization can be found in Lima and coauthors.^32^

## THEORETICAL FRAMEWORK

### The MXAN data analysis approach

The MXAN method has been proposed in the literature some years ago^3^ to derive structural information around the absorbing atom using the low energy part of the XAS spectrum, i.e., the so called XANES energy region. This method is based on the comparison between experimental data and many theoretical calculations performed by varying selected structural parameters starting from a well-defined initial geometrical configuration around the absorber. The calculation of XANES spectra is performed within the full multiple-scattering (MS) approach.^38^ The fit procedure is performed in the energy space without using any Fourier transforms algorithm, while polarized spectra can be also easily analyzed.^3^

The optimization in the space of the structural parameters is achieved using the CERN-library MINUIT^39^ routines by minimizing the square residual function, defined as

$$R_{sq}=n\frac{\sum_{i=1}^{m}w_{i}{\left[\left(y_{i}^{\text{th}}-y_{i}^{\;\text{exp}\;}\right)\varepsilon_{i}^{-1}\right]}^{2}}{\sum_{i=1}^{m}w_{i}},$$

where *n* is the number of independent parameters, *m* is the number of data points, $y_{i}^{th}$ and $y_{i}^{\;\text{exp}\;}$ are the theoretical and experimental values of the absorption, respectively, *ε_i_* is the error in each point of the experimental data set, and the statistical weight $w_{i}$ is usually set to 1. The experimental error *ε_i_* is a constant over the whole data set.

A typical fit involves an experimental energy range of about 150–200 eV from the absorption edge, and applications to several test cases indicate that the best-fit solution is quite stable and independent from the starting conditions.

The MXAN method is based on the standard MS theoretical approach within the muffin-tin (MT) approximation for the shape of the potential and the so-called “extended continuum” scheme to calculate both the continuum and the bound part of the XAS spectrum.^38^ It also uses the concept of complex optical potential based on the local density approximation of the self-energy of the excited photoelectron.^38^ The total charge density, needed to calculate the whole potential, is obtained by superimposing atomic self-consistent Hartree-Fock charges, derived using neutral or non-neutral atoms. In the MT approximation, it is necessary to define the radii of the spheres surrounding all the atoms used in the calculation, as well as the potential in the interstitial volume of the cluster. These quantities can be considered as free parameters and they must be optimized during the fit procedure. In the last version of the program, they are included in the structural loop optimization, in order to minimize the computer time and to calculate the statistical correlations with the geometrical parameters. It turns out that they are small in most of the cases with a very weak influence in the structural determination. On the other hand, it has been demonstrated that a good optimization of these non-structural parameters can minimize the errors coming from the MT approximation and overcome the need of full self-consistent potential calculation.^40^ For further details, see Refs. 1 and 3.

In order to better discriminate between different fits, it is useful to visualize the detailed behavior of the error over the whole energy range through the function $f_{e}\left(E\right)$ defined^33^ as

$$f_{e}\left(E\right)=\sqrt{{\left(y_{th}(E)-y_{\text{exp}}(E)\right)}^{2}}.$$

Here, $y_{th}\left(E\right)$ and $y_{\;\text{exp}}\left(E\right)$ are the values of the fit and experimental data at different energies. This function gives a clear indication of the goodness of different fits and is quite useful in all those cases where the difference in the residual function R_sq_ is below 10%.

### Quantum Mechanical (QM) calculations for partial charge development

At the best of our knowledge, the partial charges of the iron(II)-heme group bonded to the carbon monoxide were never developed for the CHARMM force field. The standard iron(II)-heme parameters in the CHARMM36 force field are those obtained by Kuczera *et al.*, 1990 and refer to the iron(II)-heme group bonded to the two proximal and distal histidine residues.^41^ Luthey-Schulten and coworkers optimized the partial charges and bonded parameters for the heme, hexacoordinated with ligands appropriate to Cyt *c*, relying on both protein X‐ray structures and *ab initio* density functional theory (DFT) geometry optimizations at the B3LYP/6‐31G* level.^42^ Adam and coauthors have recently carried out a similar optimization of the standard CHARMM parameters, for the Photosystem II,^43^ while Soloviov and coauthors added an *ad hoc* potential term to the CHARMM22 force field to model the ligand-heme interaction in Myoglobin–NO.^44^

Some of us, moreover, developed the carboxy Neuroglobin parameters for the Gromos force field,^45^ but it is well known that partial charges are not physical observables. Consequently, the procedure to obtain partial charges from QM calculations is slightly different in different force fields.

In order to determine the partial charges of the hexacoordinated heme in MbCO, we performed quantum chemical calculations on the isolated carboxy-imidazole iron(II) porphyrin [Fe(II)P(CO)(Im)] complex in the singlet, closed-shell electronic state. After a geometry optimization and a single-point calculation, the point charges were obtained by means of the restrained electrostatic potential (RESP) procedure,^46^ according to the CHELPG (CHarges from ELectrostatic Potentials using a Grid based method) scheme.^47^ All QM calculations were performed at B3LYP/6–311++G** level^48,49^ using the Gaussian 09 softwarepackage.^50^

### MD simulation

The starting coordinates employed for the simulations were taken from the X-ray structure of CO-bound ferrous sperm whale myoglobin at 1.15 Å resolution (Protein Data Bank, PDB 1bzr).^51^

MD simulations were performed with the GROMACS 4.6 software package^52^ using either the CHARMM36 force field^53^ or its XANES optimized version.

The XANES data of MbCO are dominated by the local geometry around the Fe absorber atom. For this reason, the refinement of the MD force field parameters was carried out by modifying the structural parameters related to the Fe surroundings. In particular, the atomic distances between the heme iron atom and both the proximal histidine nitrogen atom (Fe-N_His_) and the CO carbon atom (Fe-C_CO_), as well as the force constant of the atomic angle (N_His_-Fe-C_CO_). These force field refinements are driven by the lowering of the MXAN error function R_sq_.

Simulations were carried out at constant temperature of 300 K within a dodecahedron box using periodic boundary conditions. The P-LINCS (Parallel LINear Constraint Solver) algorithm was applied to constrain covalent bond lengths, allowing an integration step of 2 fs.^54^ A no-bond pair list cutoff of 1.2 nm was used and long range electrostatic interactions were calculated with the Particle-Mesh Ewald method (PME)^55^ using a grid spacing of 0.12 nm combined with a fourth-order B-spline interpolation to compute the potential and forces in between grid points. The temperature was controlled by separately coupling the protein and solvent to an external temperature bath.^56^ Moreover, a weak coupling for pressure was also applied.^42,57^

MD simulations were performed for 100 ns and the configurations, used for D-MXAN procedure, were collected every 5 ps.

Radial distribution functions (RdFs) are calculated with the “gmx rdf” tool of Gromacs, averaging the position of all the 20 000 MD frames. Separate calculations were carried out on the Fe atom with the CO molecule, the proximal His93 residue, and the distal His64.

### Dynamic MXAN method

The D-MXAN analysis is a method that combines Molecular Dynamic and XAS calculations, typically used to analyze disordered systems, where a quantitative XANES analysis is not feasible because of the lack of an *ab initio* reliable computational scheme. The proper configurational average spectrum is thus obtained by averaging thousands of spectra generated from distinct MD snapshots. Each snapshot is then used to generate the XANES signal associated with the corresponding instantaneous geometry, and the averaged theoretical spectrum is obtained by summing all the instantaneous spectra and dividing by the total number of used MD snapshots. Therefore, no parameter fit (neither structural nor no-structural) is carried out, with the only exception of the inelastic losses, namely, the experimental broadening, the core-hole lifetime, and the energy onset and amplitude of the plasmon contribution that are adjusted to make a quantitative comparison with the experimental data.^1,2,40^ We observe that the fitted values are the same in both cases, within the error. We obtain, for example, a value of 1.0 ± 0.44 for the core-hole lifetime in the CHARMM potential vs 1.0 ± 0.43 in the optimized one. Note that the core-hole lifetime for the K-edge of Fe is about 1.25 eV.^58^

It is important to note that, in general, in the D-MXAN analysis, we expect an *R_sq_* fitting error greater than the value normally obtained in the standard MXAN fit procedure. This is due to the impossibility in the D-MXAN procedure to optimize for each frame the overlapping factor and the interstitial potential. However, as already stated, these parameters have a very weak influence on the structural determination.^1^ The cluster size used in the D-MXAN analysis is such to include the nearest 100 atoms around the Fe absorber.

In order to calculate the total sampling length necessary to obtain a statistically significant average of the calculated XANES signal, we used the following residual function R_f_ as a function of the number of analyzed MD frames N:

$$R_{f}(N)={\left[\sum_{i}{\left[\sigma^{N}(E_{i})-\sigma^{N-1}(E_{i})\right]}^{2}\right]}^{1/2},$$

where σ^N^(E_i_) is the averaged cross section obtained using N structural frames.

## RESULTS AND DISCUSSION

### Force field parameter refinement

**FIG. 1.**
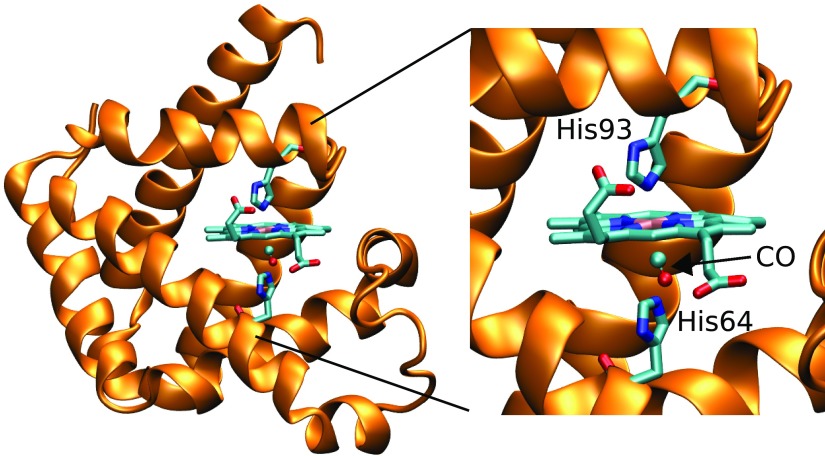
3D structure of the myoglobin protein bonded to carbon monoxide (MbCO; PDB id 1A6G). In the inset, an enlargement of the heme molecule. Lateral chains of proximal (His93) and distal (His64) histidine are highlighted.

The Carboxy-Myoglobin is a challenging system for the D-MXAN analysis because there is a high number of parameters that controls the atomic configurations around the Fe atom generated by the MD simulation in its time window. The Fe atom is embedded in the heme group (see Fig. 1) and strongly interacts with the CO molecule and with the proximal histidine (His93), while other residues can also influence the heme conformation, first of all the distal histidine (His64).

The standard iron(II)-heme parameters in the CHARMM36 force field^33^ refer to the deoxy Mb, where the iron(II)-heme group is bonded to the two proximal and distal histidine residues. Therefore, we first carried out standard QM calculations to produce a set of atomic charges for MbCO, compatible with the CHARMM36 force field (see Theoretical Framework section for details). In Table S2, we report the calculated and original charges for the heme group. Note that the total −2 negative charge is now distributed on the heme group, the proximal histidine, and the CO molecule.

Then, we carried out an iterative procedure that evaluated the effect of different force field parameters on the D-MXAN results, starting from those playing a major effect on the XANES signal. Overall, we optimized the bond parameters for the heme group, the CO molecule, and the proximal histidine His93, the force constant of the Fe-CO angle. In Table S1, we report the original CHARMM and our optimized force field parameters (also made available in GROMACS format as supplementary material).

**FIG. 2.**
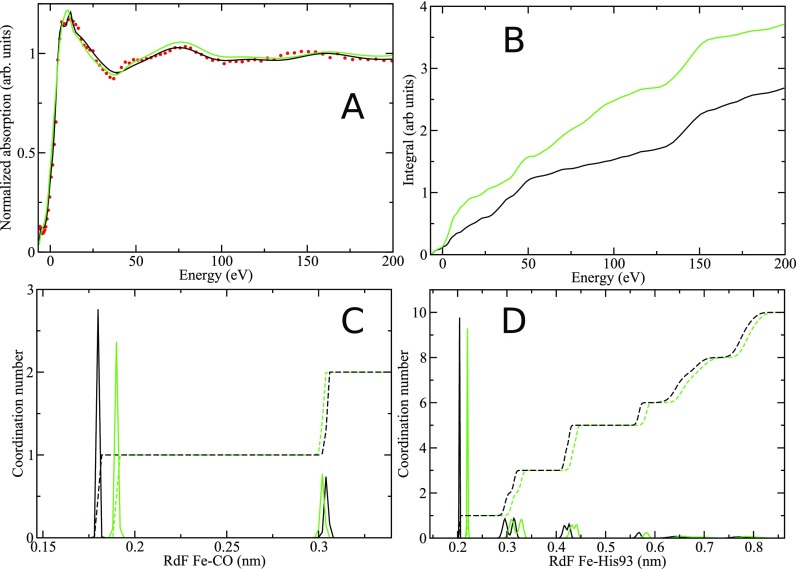
Comparison between our optimized potential and the standard CHARMM potential. (**a**) D-MXAN fit with the optimized potential (black full line) and the standard CHARMM one (green color). Experimental data are shown as red points; (**b**) integrated area of the fit error function f(E) in normalized area units; (**c**) RdF (full line) and coordination number (dashed line) between the Fe atom and the CO molecule; and (**d**) RdF (full line) and coordination number (dashed line) between the Fe atom and the proximal His93 residue.

We show in Fig. 2(a) the comparison between the experimental data (red points), two D-MXAN fit performed using the standard CHARMM36 force field (green line), and our optimized force field (black line). The CHARMM curve is able to reproduce the overall shape of the experimental data, but there are several discrepancies in the whole energy range. Here, the R_sq_ fitting error is 3.99, which is a relatively large value for the standard MXAN fit but can be considered as a good starting point for the D-MXAN analysis.

In the case of the D-MXAN fit obtained with the optimized potential, the R_sq_ error reduces to 1.95 and now the agreement with the experimental data is rather good in the whole energy range. It should be noted, however that the MXAN R_sq_ obtained in the static fit is 1.71. This is not surprisingly because in the case of static MXAN fit, we were able to optimize both structural and non-structural parameters. In panel B, we report the behavior of the integral of $f_{e}\left(E\right)$ as function of energy. The difference between the two curves is big over the whole energy range, confirming the great improvement of the quality of the fit using the optimized force field parameters. Finally, convergence of both calculations was evaluated by plotting of the residual function *R_f_* as a function of the number of MD frames. Figure S2 (supplementary material) shows that after 200 frames *R_f_* is always below 10^−6^ for both CHARMM and the optimized potentials.

In the following, we show the structural parameters that have the strongest influence on the D-MXAN fits. In Fig. 2(c), we report the comparison between the Fe-CO radial distribution function (RdF) obtained with the original CHARMM36 force field (green color) and our optimized potential (black color). Our potential optimization reduces the distance between the Fe atom and the C atom of the CO molecule from 1.90 Å to 1.80 Å (first peak) and increases the Fe-O distance from 3.02 to 3.04 Å (second peak). It is notable the opposite behavior of the two peaks in the RdF. This effect is due to the increase in the C-O bond length from 1.128 Å in the standard CHARMM36 force field to 1.25 Å in our optimized potential. In fact, standard CHARMM36 parameters model a photodissociated CO molecule, while our refined model, as already stated, deals with the covalent bonded MbCO complex in which the CO distance is longer. Note that the adjustment of the C-O bond length can have a negligible impact on the long distance properties of the protein, but we have demonstrated that it produces a bad reproduction of the XANES signal, being responsible for the greater part of the Rsq error difference between the CHARMM potential and our optimized one.

Our optimized value of the Fe-C distance, as already mentioned, reduces the distance between the Fe atom and the C atom of the CO molecule from 1.90 Å to 1.80 Å (see Table S1). This is in good agreement with both XRD and static MXAN fit determination. In fact, XRD indicates a value from 1.73 Å (PDB id 1BZR^51^) to 1.82 Å (PDB id 1A6G^59^) for the Fe-CO distance, depending on the experimental conditions. The static MXAN fits performed on single crystal and in water solution give a value of 1.83 ± 0.02 Å and 1.83 ± 0.01 Å, respectively.^4,32^

Our optimization procedure also produced a slight decrease in the Fe-C-O bending constant from 70.0 (CHARMM36) to 67.4 kcal/mol rad^2^ (our optimized potential). Note that although the force field equilibrium angle value (180°), the sampled simulation value is 172.9° ± 3.5 in both cases, because of the additive effects of different force field parameters. This value is close to experimental determination in liquid solution (171.12°)^32^ and to the X-ray crystallographic (XRD) value which ranges between 171° and 173°.^51,59^ The effect of the potential optimization results also in a change of the Fe-His93 RdF [first peak distances in Fig. 2(d)] from 2.20 to 2.04 Å. This last value is in good agreement with what found in the XRD analysis and static MXAN fits performed on crystal and water solution.^4,32,51,59^ In Table S1 (supplementary material) we report, a summary of the changes of the above parameters changed during the potential optimization.

Atomic charges of heme group, CO molecule, and proximal His93 have been *ab initio* calculated from us. The values are reported in Table S2 (supplementary material) and are different from the original data of CHARMM36 that are also reported for comparison in the same table.

The above described small changes in the MbCO force field parameters were enough to yield a better reproduction of the XANES experimental signal on the whole energy range using a pure MD sampling.

It is also worth noting that the refining procedure is manual and iterative. Changes in the force field parameters that produced an increase in the R_sq_ error were hence discarded.

### D-MXAN variability during MD sampling

**FIG. 3.**
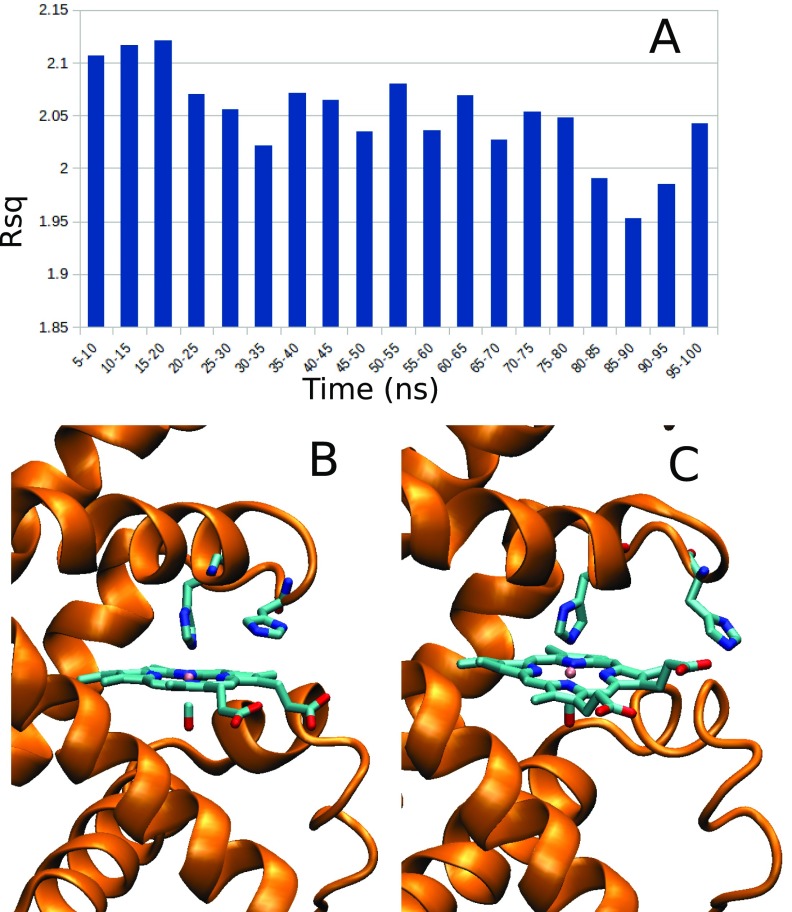
(**a**) R_sq_ values in 5 ns time window during the 100 ns MD simulation. The first 5 ns of simulation has been discarded as equilibration time. Representative structures visited during the simulation with our optimized potential. (**b**) In frames with relatively “big R_sq_,” His97 samples mainly conformation inner to the heme ring. (**c**) In the frames with relatively “small R_sq_,” His97 visits only conformations far from the heme ring. Multimedia views: https://doi.org/10.1063/1.5031806.1;
10.1063/1.5031806.1
https://doi.org/10.1063/1.5031806.2
10.1063/1.5031806.2

Usually R_sq_ converges after few nanoseconds of MD simulation but in the case of the Myoglobin, we observe a relatively high variability of this value along the MD simulation time. To see this effect, we report in Fig. 3(a) the R_sq_ value in time window of 5 ns for the whole 100 ns MD simulation. The R_sq_ in single time windows goes from 2.121 in the 15–20 ns time window to 1.953 in the 85–90 ns time window. The total R_sq_ average obtained using the whole 5–100 ns MD simulation is 2.046. The relatively great variability shows also a specific trend, with greater R_sq_ values in the first 20 ns of simulation. In the supplementary material, we have investigated this issue with more detail. In Fig. S1(a), we plot the comparison between the experimental data (red points) and the two D-MXAN fit performed using our optimized force field in the best and worse time windows (in brown and cyan colors, respectively). Enlargement in Fig. S1(b) shows how the brown curve, corresponding to the 85–90 ns time window with Rsq = 1.953, is particularly closer to the experimental data in the 5–8 eV range. In line, the fit error function f(E), in Fig. S1(c) shows the separation of the two curves in that energy range. The difference then is maintained nearly constant in the whole energy range.

We further investigated this behavior and we found that the most significant difference in the “small R_sq_” and “big R_sq_” MD frames was a different sampling of the His97 residue, the third farther histidine from Fe, after the proximal His93 and the distal His64. In particular, in the “big R_sq_” frames, His97 samples mainly conformation inner to the heme ring [see a representative structure in Fig. 3(b)], with a minority of conformations being outer of the heme ring. The “small R_sq_,” on the contrary, visits only conformations with His97 farthest from the heme ring [see a representative structure in Fig. 3(c)].

The fact that His97 visits different conformational basins is somehow normal in a MD simulation and, actually, it could be related to the observed role played by this residue in the stability of the heme group in Myoglobin.^60^ The interesting results, coming from this D-MXAN analysis, are that the sampling for a metalloprotein should be of the order of tens of nanoseconds for such type of applications.

### D-MXAN sensitivity to different MD parameters

**FIG. 4.**
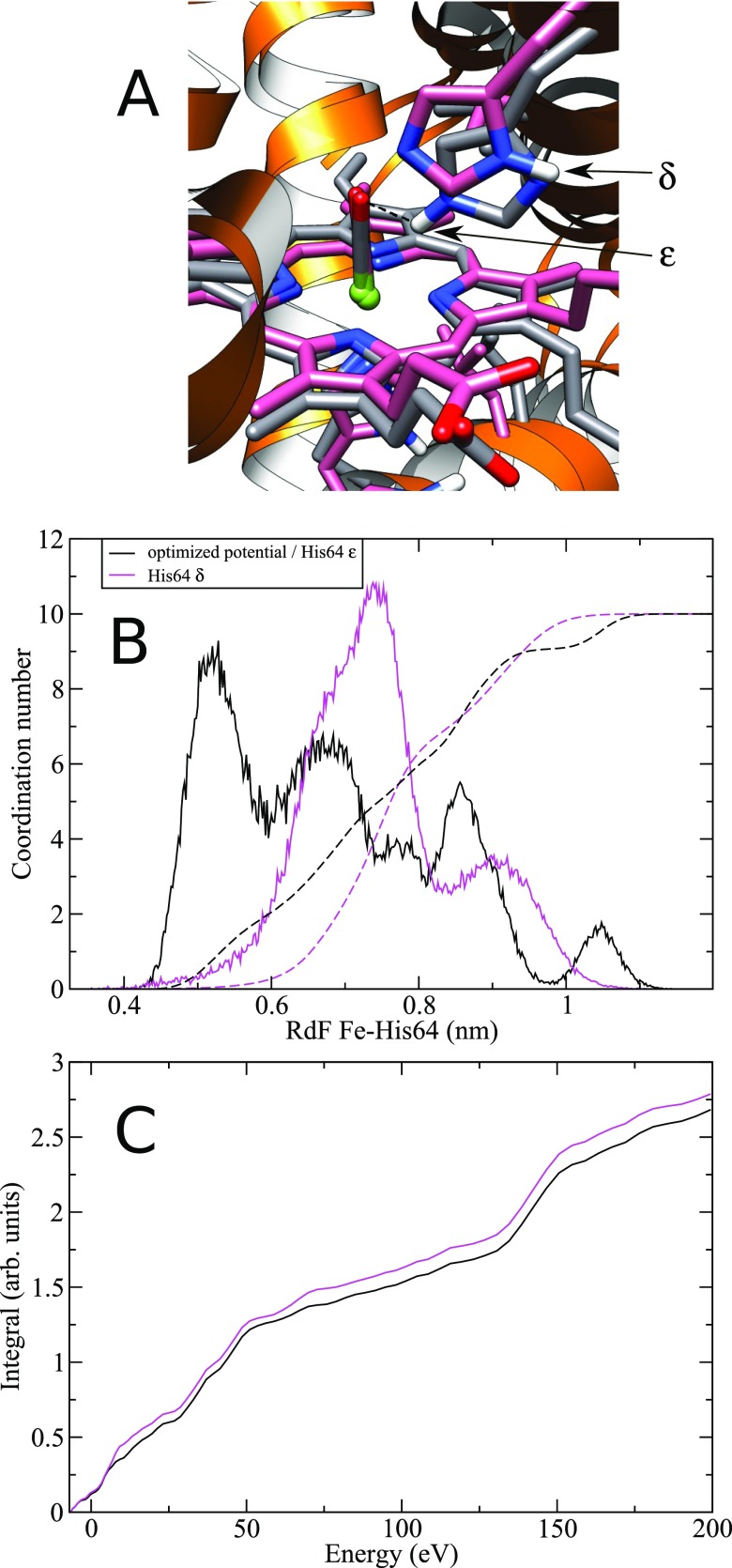
Comparison between the ε (black color) and δ nitrogen (magenta color) protonated distal His64. (**a**) 3D snapshot of the two His64 forms. The hydrogen bond between His64 and the CO molecule is shown with a dashed line; (**b**) RdF (full line) and coordination number (dashed line) between the Fe atom and the His64 residue; and (**c**) integrated area of the fit error function f(E) in normalized area units.

In order to demonstrate the sensitivity of the D-MXAN method to small structural details, we carried out a MD simulation in which the His64 hydrogen atom was on the δ nitrogen instead of the preferential ε one [see Fig. 4(a)]. Only this last conformation permits the formation of a hydrogen bond between the distal histidine His64 and the CO molecule [dashed line in Fig. 4(a)].^24,61,62^ The structural rearrangements around the Fe absorber atom, following the loss of the hydrogen bond in the His64 δ nitrogen tautomer, are shown in Fig. 4(b). The Fe-His64 distance changes from 5.1 to 7.4 Å [first RdF peak distance in Fig. 4(b)] and at the same time it samples a smaller distance range as compared to the preferential ε nitrogen, being the great majority of conformations at a range distance of 6–8 Å from Fe.

The described alterations in the MD sampling of the δ nitrogen tautomer produce an increase in the D-MXAN Rsq value up to 2.16 from the 1.95 of the preferential ε nitrogen tautomer. The increase is significant and it is also demonstrated by the behavior of the integral of the $f_{e}\left(E\right)$ reported in Fig. 4(c). The black line is related to the preferential ε tautomer and it is always below the corresponding values of the δ tautomer.

## CONCLUSIONS

In this paper, we have reported a detailed D-MXAN analysis of the Fe K-edge of CO Myoglobin in water solution. The quantitative comparison between experimental and computational data is a powerful tool to investigate and improve the quality of classical force field parameters and the sensitivity of the D-MXAN method is enough to highlight the structural and dynamical details at a resolution that is difficult to reach with other experimental methods.

We have developed a new set of force field parameters for the CO Myoglobin protein in the water solution, capable of correcting the greatest part of the structural discrepancy between classical MD sampling and experimental determinations. This is quite important in all those cases where a detailed analysis of the heme properties is performed.

## SUPPLEMENTARY MATERIAL

See supplementary material for optimized bonded parameters and atomic charges in our modified potential vs the original CHARMM36; for D-MXAN fits on two of the 5 ns time windows; and for the plot of the residual function *R_f_* as a function of the number of MD frames N.

## References

[1] M. Benfatto , E. Pace , N. Sanna , C. Padrin , and G. Chillemi , “MXAN and molecular dynamics: A new way to look to the XANES (X-ray absorption near edge structure) energy region,” in *Multiple Scattering Theory for Spectroscopies*, edited by Sébilleau D., Hatada K., and Ebert H. ( Springer International Publishing, 2018), Vol. 204, pp. 197–219.

[2] M. Benfatto and C. A. Meneghini , “Close look into the low energy region of the XAS spectra: The XANES region,” in *Synchrotron Radiation*, edited by Mobilio S., Boscherini F., and Meneghini C. ( Springer, Berlin, Heidelberg, 2015), pp. 213–240.

[3] M. Benfatto and S. Della Longa , “Geometrical fitting of experimental XANES spectra by a full multiple-scattering procedure,” J. Synchrotron Radiat. 8, 1087–1094 (2001). 10.1107/S0909049501006422 11486360

[4] S. D. Longa , A. Arcovito , M. Girasole , J. L. Hazemann , and M. Benfatto , “Quantitative analysis of X-ray absorption near edge structure data by a full multiple scattering procedure: The Fe-CO geometry in photolyzed carbonmonoxy-myoglobin single crystal,” Phys. Rev. Lett. 87, 155501 (2001). 10.1103/PhysRevLett.87.155501 11580707

[5] A. Arcovito *et al.*, “X-ray structure analysis of a metalloprotein with enhanced active-site resolution using *in situ* x-ray absorption near edge structure spectroscopy,” Proc. Natl. Acad. Sci. U. S. A. 104, 6211–6216 (2007). 10.1073/pnas.0608411104 17404234 PMC1851025

[6] R. Sarangi *et al.*, “The x-ray absorption spectroscopy model of solvation about sulfur in aqueous L-cysteine,” J. Chem. Phys. 137, 205103 (2012). 10.1063/1.4767350 23206038 PMC3526153

[7] E. Antonini and M. Brunori , *Hemoglobin and Myoglobin in Their Reactions with Ligands* ( North-Holland, Amsterdam, 1971), 436 pp.

[8] H. Frauenfelder , B. H. McMahon , and P. W. Fenimore , “Myoglobin: The hydrogen atom of biology and a paradigm of complexity,” Proc. Natl. Acad. Sci. U. S. A. 100, 8615–8617 (2003). 10.1073/pnas.1633688100 12861080 PMC166357

[9] J. S. Olson *et al.*, “The role of the distal histidine in myoglobin and haemoglobin,” Nature 336, 265–266 (1988). 10.1038/336265a0 3057383

[10] M. F. Perutz and F. S. Mathews , “An X-ray study of azide methaemoglobin,” J. Mol. Biol. 21, 199–202 (1966). 10.1016/0022-2836(66)90088-X 5969763

[11] M. Brunori , “Myoglobin strikes back,” Protein Sci. 19, 195–201 (2010). 10.1002/pro.300 19953516 PMC2865719

[12] J. B. Wittenberg , “Myoglobin function reassessed,” J. Exp. Biol. 206, 2011–2020 (2003). 10.1242/jeb.00243 12756283

[13] A. Tomita , U. Kreutzer , S.-i. Adachi , S.-y. Koshihara , and T. Jue , “ ‘ It's hollow': The function of pores within myoglobin,” J. Exp. Biol. 213, 2748–2754 (2010). 10.1242/jeb.042994 20675544

[14] C. Bossa *et al.*, “Extended molecular dynamics simulation of the carbon monoxide migration in sperm whale myoglobin,” Biophys. J. 86, 3855–3862 (2004). 10.1529/biophysj.103.037432 15189882 PMC1304287

[15] G. Hummer , F. Schotte , and P. A. Anfinrud , “Unveiling functional protein motions with picosecond x-ray crystallography and molecular dynamics simulations,” Proc. Natl. Acad. Sci. U. S. A. 101, 15330–15334 (2004). 10.1073/pnas.0405295101 15489270 PMC523450

[16] D. R. Nutt and M. Meuwly , “CO migration in native and mutant myoglobin: Atomistic simulations for the understanding of protein function,” Proc. Natl. Acad. Sci. U. S. A. 101, 5998–6002 (2004). 10.1073/pnas.0306712101 15067128 PMC395912

[17] L. Maragliano , G. Cottone , G. Ciccotti , and E. Vanden-Eijnden , “Mapping the network of pathways of CO diffusion in myoglobin,” J. Am. Chem. Soc. 132, 1010–1017 (2010). 10.1021/ja905671x 20039718

[18] R. Elber and M. Karplus , “Multiple conformational states of proteins: A molecular dynamics analysis of myoglobin,” Science 235, 318–321 (1987). 10.1126/science.3798113 3798113

[19] M. Anselmi , A. Di Nola , and A. Amadei , “The kinetics of ligand migration in crystallized myoglobin as revealed by molecular dynamics simulations,” Biophys. J. 94, 4277–4281 (2008). 10.1529/biophysj.107.124529 18310235 PMC2480659

[20] L. U. L. Brinkmann and J. S. Hub , “Ultrafast anisotropic protein quake propagation after CO photodissociation in myoglobin,” Proc. Natl. Acad. Sci. U. S. A. 113, 10565–10570 (2016). 10.1073/pnas.1603539113 27601659 PMC5035865

[21] T. R. M. Barends *et al.*, “Direct observation of ultrafast collective motions in CO myoglobin upon ligand dissociation,” Science 350, 445–450 (2015). 10.1126/science.aac5492 26359336

[22] D. R. Nutt and M. Meuwly , “Theoretical investigation of infrared spectra and pocket dynamics of photodissociated carbonmonoxy myoglobin,” Biophys. J. 85, 3612–3623 (2003). 10.1016/S0006-3495(03)74779-1 14645054 PMC1303666

[23] D. R. Nutt and M. Meuwly , “Ligand dynamics in myoglobin: Calculation of infrared spectra for photodissociated NO,” ChemPhysChem 5, 1710–1718 (2004). 10.1002/cphc.200400220 15580931

[24] M. Meuwly , “On the influence of the local environment on the CO stretching frequencies in native myoglobin: Assignment of the B-states in MbCO,” ChemPhysChem 7, 2061–2063 (2006). 10.1002/cphc.200600304 16955519

[25] M. Anselmi , M. Aschi , A. Di Nola , and A. Amadei , “Theoretical characterization of carbon monoxide vibrational spectrum in sperm whale myoglobin distal pocket,” Biophys. J. 92, 3442–3447 (2007). 10.1529/biophysj.106.098442 17307822 PMC1853160

[26] C. Oostenbrink , A. Villa , A. E. Mark , and W. F. Van Gunsteren , “A biomolecular force field based on the free enthalpy of hydration and solvation: The GROMOS force-field parameter sets 53A5 and 53A6,” J. Comput. Chem. 25, 1656–1676 (2004). 10.1002/jcc.20090 15264259

[27] C. Kiefl *et al.*, “Heme distortions in sperm-whale carbonmonoxy myoglobin: Correlations between rotational strengths and heme distortions in MD-generated structures,” J. Am. Chem. Soc. 124, 3385–3394 (2002). 10.1021/ja011961w 11916424

[28] E. R. Henry , M. Levitt , and W. A. Eaton , “Molecular dynamics simulation of photodissociation of carbon monoxide from hemoglobin,” Proc. Natl. Acad. Sci. U. S. A. 82, 2034–2038 (1985). 10.1073/pnas.82.7.2034 3856881 PMC397485

[29] J. W. Petrich *et al.*, “Ligand binding and protein relaxation in heme proteins: A room temperature analysis of nitric oxide geminate recombination,” Biochemistry (Mosc.) 30, 3975–3987 (1991). 10.1021/bi00230a025 2018766

[30] D. A. Case and M. Karplus , “Stereochemistry of carbon monoxide binding to myoglobin and hemoglobin,” J. Mol. Biol. 123, 697–701 (1978). 10.1016/0022-2836(78)90214-0 691060

[31] X. Y. Li and T. G. Spiro , “Is bound carbonyl linear or bent in heme proteins? Evidence from resonance Raman and infrared spectroscopic data,” J. Am. Chem. Soc. 110, 6024–6033 (1988). 10.1021/ja00226a017 22148777

[32] F. A. Lima *et al.*, “Probing the electronic and geometric structure of ferric and ferrous myoglobins in physiological solutions by Fe K-edge absorption spectroscopy,” Phys. Chem. Chem. Phys. 16, 1617–1631 (2014). 10.1039/C3CP53683A 24317683

[33] G. Chillemi , E. Pace , M. D'Abramo , and M. Benfatto , “Equilibrium between 5- and 6-fold coordination in the first hydration shell of Cu(II),” J. Phys. Chem. A 120, 3958–3965 (2016). 10.1021/acs.jpca.6b03569 27195961

[34] M. Antalek *et al.*, “Solvation structure of the halides from x-ray absorption spectroscopy,” J. Chem. Phys. 145, 044318 (2016). 10.1063/1.4959589 27475372 PMC4967075

[35] P. D'Angelo , O. M. Roscioni , G. Chillemi , S. Della Longa , and M. Benfatto , “Detection of second hydration shells in ionic solutions by XANES: Computed spectra for Ni^2+^ in water based on molecular dynamics,” J. Am. Chem. Soc. 128, 1853–1858 (2006). 10.1021/ja0562503 16464084

[36] P. D'Angelo *et al.*, “Dynamic investigation of protein metal active sites: Interplay of XANES and molecular dynamics simulations,” J. Am. Chem. Soc. 132, 14901–14909 (2010). 10.1021/ja1056533 20919711

[37] G. Chillemi *et al.*, “Carbon monoxide binding to the heme group at the dimeric interface modulates structure and copper accessibility in the Cu, Zn superoxide dismutase from Haemophilus ducreyi: *In silico* and *in vitro* evidences,” J. Biomol. Struct. Dyn. 30, 269–279 (2012). 10.1080/07391102.2012.680028 22686457

[38] T. A. Tyson , K. O. Hodgson , C. R. Natoli , and M. Benfatto , “General multiple-scattering scheme for the computation and interpretation of x-ray-absorption fine structure in atomic clusters with applications to SF_6_, GeCl_4_, and Br_2_ molecules,” Phys. Rev. B 46, 5997–6019 (1992). 10.1103/PhysRevB.46.5997 10002283

[39] F. James and M. Roos , “Minuit - A system for function minimization and analysis of the parameter errors and correlations,” Comput. Phys. Commun. 10, 343–367 (1975). 10.1016/0010-4655(75)90039-9

[40] M. Benfatto and S. D. Longa , “MXAN: New improvements for potential and structural refinement,” J. Phys.: Conf. Ser. 190, 012031 (2009). 10.1088/1742-6596/190/1/012031

[41] K. Kuczera , J. Kuriyan , and M. Karplus , “Temperature dependence of the structure and dynamics of myoglobin,” J. Mol. Biol. 213, 351–373 (1990). 10.1016/S0022-2836(05)80196-2 2342112

[42] F. Autenrieth , E. Tajkhorshid , J. Baudry , and Z. Luthey-Schulten , “Classical force field parameters for the heme prosthetic group of cytochrome *c*,” J. Comput. Chem. 25, 1613–1622 (2004). 10.1002/jcc.20079 15264255

[43] S. Adam , M. Knapp-Mohammady , J. Yi , and A.-N. Bondar , “Revised CHARMM force field parameters for iron-containing cofactors of photosystem II,” J. Comput. Chem. 39, 7–20 (2018). 10.1002/jcc.24918 28850168

[44] M. Soloviov , A. K. Das , and M. Meuwly , “Structural interpretation of metastable states in myoglobin-NO,” Angew. Chem., Int. Ed. 55, 10126–10130 (2016). 10.1002/anie.201604552 27410027

[45] M. Anselmi , M. Brunori , B. Vallone , and A. Di Nola , “Molecular dynamics simulation of deoxy and carboxy murine neuroglobin in water,” Biophys. J. 93, 434–441 (2007). 10.1529/biophysj.106.099648 17468165 PMC1896225

[46] C. I. Bayly , P. Cieplak , W. Cornell , and P. A. Kollman , “A well-behaved electrostatic potential based method using charge restraints for deriving atomic charges: The RESP model,” J. Phys. Chem. 97, 10269–10280 (1993). 10.1021/j100142a004

[47] C. M. Breneman and K. B. Wiberg , “Determining atom-centered monopoles from molecular electrostatic potentials. The need for high sampling density in formamide conformational analysis,” J. Comput. Chem. 11, 361–373 (1990). 10.1002/jcc.540110311

[48] A. D. Becke , “Density‐functional thermochemistry. III. The role of exact exchange,” J. Chem. Phys. 98, 5648–5652 (1993). 10.1063/1.464913

[49] C. Lee , W. Yang , and R. G. Parr , “Development of the Colle-Salvetti correlation-energy formula into a functional of the electron density,” Phys. Rev. B 37, 785–789 (1988). 10.1103/PhysRevB.37.785 9944570

[50] M. J. Frisch *et al.*, *Gaussian 09, Revision D.01* ( Gaussian, Inc, Wallingford, CT, 2016).

[51] G. S. Kachalova , A. N. Popov , and H. D. Bartunik , “A steric mechanism for inhibition of CO binding to heme proteins,” Science 284, 473–476 (1999). 10.1126/science.284.5413.473 10205052

[52] S. Pronk *et al.*, “GROMACS 4.5: A high-throughput and highly parallel open source molecular simulation toolkit,” Bioinformatics 29, 845–854 (2013). 10.1093/bioinformatics/btt055 23407358 PMC3605599

[53] R. B. Best *et al.*, “Optimization of the additive CHARMM all-atom protein force field targeting improved sampling of the backbone ϕ, ψ and side-chain χ_1_ and χ_2_ dihedral angles,” J. Chem. Theory Comput. 8, 3257–3273 (2012). 10.1021/ct300400x 23341755 PMC3549273

[54] B. P. Hess , “LINCS: A parallel linear constraint solver for molecular simulation,” J. Chem. Theory Comput. 4, 116–122 (2008). 10.1021/ct700200b 26619985

[55] T. Darden , D. York , and L. Pedersen , “Particle mesh Ewald: An N ⋅log (N) method for Ewald sums in large systems,” J. Chem. Phys. 98, 10089–10092 (1993). 10.1063/1.464397

[56] G. Bussi , D. Donadio , and M. Parrinello , “Canonical sampling through velocity rescaling,” J. Chem. Phys. 126, 014101 (2007). 10.1063/1.2408420 17212484

[57] H. J. C. Berendsen , J. P. M. Postma , W. F. van Gunsteren , A. DiNola , and J. R. Haak , “Molecular dynamics with coupling to an external bath,” J. Chem. Phys. 81, 3684–3690 (1984). 10.1063/1.448118

[58] M. O. Krause and J. H. Oliver , “Natural widths of atomic K and L levels, K α X‐ray lines and several K L L Auger lines,” J. Phys. Chem. Ref. Data 8, 329–338 (1979). 10.1063/1.555595

[59] J. Vojtěchovský , K. Chu , J. Berendzen , R. M. Sweet , and I. Schlichting , “Crystal structures of myoglobin-ligand complexes at near-atomic resolution,” Biophys. J. 77, 2153–2174 (1999). 10.1016/S0006-3495(99)77056-6 10512835 PMC1300496

[60] L. Tofani *et al.*, “Spectroscopic and interfacial properties of myoglobin/surfactant complexes,” Biophys. J. 87, 1186–1195 (2004). 10.1529/biophysj.104.041731 15298921 PMC1304457

[61] J. B. Asbury , T. Steinel , and M. D. Fayer , “Hydrogen bond networks: Structure and evolution after hydrogen bond breaking,” J. Phys. Chem. B 108, 6544–6554 (2004). 10.1021/jp036600c

[62] K. Nienhaus , J. S. Olson , S. Franzen , and G. U. Nienhaus , “The origin of stark splitting in the initial photoproduct state of MbCO,” J. Am. Chem. Soc. 127, 40–41 (2005). 10.1021/ja0466917 15631438

